# Pairwise difference regressions are just weighted averages

**DOI:** 10.1038/s41598-021-02096-3

**Published:** 2021-11-29

**Authors:** Carlos Góes

**Affiliations:** Department of Economics, University of California, San Diego, USA

**Keywords:** Statistics, Psychology and behaviour

**arising from**: R. F. Savaris et al.; *Scientific Reports* 10.1038/s41598-021-84092-1 (2021).

Savaris et al.^1^ aim at “verifying if staying at home had an impact on mortality rates.” This short note shows that the methodology they have applied in their paper does not allow them to do so. An estimated coefficient $\beta \approx 0$ does not imply that there is no association between the variables in either country. Rather, their pairwise difference regressions are computing coefficients that are weighted-averages of region-specific time series regressions, such that **it is possible that the association is significant in both regions but their weighted-average is close to zero**. Therefore, the results do not back up the conclusions of the paper.

Consider two regions: *A* and *B*. Suppose that the true relationships between the change in deaths per million $(\Delta Y_t^i)$ and the change in an index of staying at home $(\Delta X_t^i)$ at epidemiological week *t* in countries $i=A,B$ are the following:

$$\begin{aligned} \Delta Y_t^A= & {} \beta _A \Delta X_t^A + \varepsilon _t^A \\ \Delta Y_t^B= & {} \beta _B \Delta X_t^B + \varepsilon _t^B \end{aligned}$$

For simplicity in exposition, assume that $\Delta X_t^A, \Delta X_t^B, \varepsilon _t^A, \varepsilon _t^B$ are all zero mean, iid processes. By subtracting the second equation from the first and defining $\Delta Y_t \equiv \Delta Y_t^A - \Delta Y_t^B$ and $\Delta X_t \equiv \Delta X_t^A - \Delta X_t^B$, we can write:

1 $$\begin{aligned} \Delta Y_t^A - \Delta Y_t^B= & {} \beta ( \Delta X_t^A - \Delta X_t^B) + (\beta _A - \beta ) \Delta X_t^A - (\beta _B-\beta ) \Delta X_t^B + (\varepsilon _t^A - \varepsilon _t^B) \nonumber \\ \Delta Y_t= & {} \beta \Delta X_t + \eta _t \end{aligned}$$

where $\eta _t \equiv (\beta _A - \beta ) \Delta X_t^A - (\beta _B-\beta ) \Delta X_t^B + (\varepsilon _t^A - \varepsilon _t^B)$. It is easy to see that, for $\beta _i \ne \beta$, estimation of $\beta$ will not be consistent, since, by construction, $cov(\Delta X_t,\eta _t) \ne 0$.

If nonetheless one estimates (1) by ordinary least squares, what does the regression coefficient $\beta$ converge to? It turns out that it converges to a variance-weighted average of $\beta _A$, $\beta _B$, as summarized in the following proposition.

## Proposition 1

Let $\Delta X_t^A, \Delta X_t^B, \varepsilon _t^A, \varepsilon _t^B, \beta _A$, $\beta _B, \beta$ be all as above. Then ${\hat{\beta }}$, the ordinary least squares coefficient of regressing $\Delta Y_t$ on $\Delta X_t$, converges in probability to:

2 $$\begin{aligned} \beta = w \beta _A + (1-w) \beta _B \end{aligned}$$

with $$w \equiv \frac{{\mathbb {E}}[(\Delta X_t^A)^2]}{{\mathbb {E}}[(\Delta X_t^A)^2] + {\mathbb {E}}[(\Delta X_t^B) ^2] }$$.

## Proof

Under the stated assumptions, ${\hat{\beta }} = \frac{\sum _{t}^T \Delta X_t \Delta Y_t}{\sum _{t}^T \Delta X_t^2} \xrightarrow {p} \frac{ {\mathbb {E}}[\Delta Y_t \Delta X_t] }{{\mathbb {E}}[\Delta X_t^2] } \equiv \beta$. One can calculate the population parameter $\beta$ analytically:

$$\begin{aligned} \beta= & {} \frac{ {\mathbb {E}}[\Delta Y_t \Delta X_t] }{{\mathbb {E}}[\Delta X_t^2] } \\= & {} \frac{ {\mathbb {E}}[ ( \Delta Y_t^A - \Delta Y_t^B)( \Delta X_t^A - \Delta X_t^B)] }{{\mathbb {E}}[( \Delta X_t^A - \Delta X_t^B)^2] } \\= & {} \frac{ {\mathbb {E}}[ \Delta Y_t^A \Delta X_t^A] + {\mathbb {E}}[ \Delta Y_t^B \Delta X_t^B] }{{\mathbb {E}}[(\Delta X_t^A)^2] + {\mathbb {E}}[(\Delta X_t^B) ^2] } \qquad \because {\mathbb {E}}[\Delta X_t^A \Delta X_t^B] = {\mathbb {E}}[\Delta X_t^A \Delta Y_t^B] = {\mathbb {E}}[\Delta X_t^B \Delta Y_t^A] = 0 \\= & {} \frac{{\mathbb {E}}[(\Delta X_t^A)^2]}{{\mathbb {E}}[(\Delta X_t^A)^2] + {\mathbb {E}}[(\Delta X_t^B) ^2] } \frac{ {\mathbb {E}}[ \Delta Y_t^A \Delta X_t^A]}{ {\mathbb {E}}[(\Delta X_t^A)^2] } + \frac{{\mathbb {E}}[(\Delta X_t^B)^2]}{{\mathbb {E}}[(\Delta X_t^A)^2] + {\mathbb {E}}[(\Delta X_t^B) ^2] } \frac{{\mathbb {E}}[ \Delta Y_t^B \Delta X_t^B]}{ {\mathbb {E}}[(\Delta X_t^B)^2]} \end{aligned}$$

Note that $\frac{ {\mathbb {E}}[ \Delta Y_t^A \Delta X_t^A]}{ {\mathbb {E}}[(\Delta X_t^A)^2] } = \beta _A$ and $\frac{ {\mathbb {E}}[ \Delta Y_t^B \Delta X_t^B]}{ {\mathbb {E}}[(\Delta X_t^B)^2] } = \beta _B$. Using that and the definition of *w* we arrive at the desired result. $\square$

The intuition regarding the (2) in the Proposition is simple. Whenever the variance of $\Delta X_t^A$ is large relative to country *B*, $w \rightarrow 1$ and $\beta \rightarrow \beta _A$. Similarly, if the variance of $\Delta X_t^B$ is large relative to country *A*, $w \rightarrow 0$ and $\beta \rightarrow \beta _B$.

What does this mean for the analysis of Savaris *et al.*^1^? In general, it means that one cannot interpret their estimated ${\hat{\beta }}$ without knowing the underlying relative variances. Additionally, one cannot infer that an insignificant (or even numerically zero) ${\hat{\beta }}$ implies absence of association in either country.

To see that, suppose countries *A* and *B* have identical variance in their independent variables, but $\beta _A$, $\beta _B$ are different. In country *A*, the policymaker adjusts stay-at-home orders in response to the increase in deaths, such that the change in the percentage of the public staying at home is positively correlated with the change in deaths. In country *B*, the policymaker does not act, such that the change in share of population staying at home is negatively correlated with contacts, infections, and deaths.

**Figure 1:**
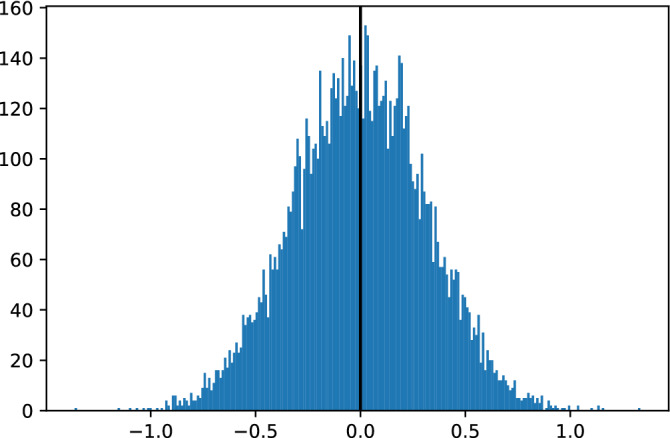
In-sample simulated ${\hat{\beta }}$ for 10,000 random draws with $\Delta X_t^i \sim N(0,10)$, $\varepsilon _t^i \sim N(0,1)$, and $\Delta Y_t^i = \beta _i \Delta X_t^i + \varepsilon ^i_t$, for $i = A, B$; $T=1,000$; and $\beta _A =10$, $\beta _B = -10$. As expected the sample values are distributed around the true population value of $\beta =0$.

Consider the case in which $\beta _B = - \beta _A$. Then, since the regions have identical variance, $w=1/2$ and $\beta = 0$ even though the true association is nonzero in both countries. The regression coefficients in Savaris *et al.*^1^ should not lead one to conclude that, in either country, there is no association between the change in mobility and the change in deaths per million. Figure 1 shows the result of 10,000 simulated $\hat{\beta}$ in which $\beta _A = 10$ and $\beta _B = -10$. In this case, $var(X^A_t) = var(X^B_t)$ and variables are iid and normally distributed. As expected, sample estimates are distributed around the population value of $\beta =0$.

For $\beta _A \ne \beta _B$, then, region-specific dynamics are heterogeneous and, as shown by Pesaran & Smith^2^, aggregating or pooling slopes can lead to biased estimates, making individual regressions for each group member preferable. If authors assume that $\beta _A = \beta _B$ for each pair in their sample – i.e., homogeneous $\beta$ –, then dynamic panels would have many advantages in terms of efficiency and use of instruments to circumvent endogeneity. In either case, their pairwise approach would not be appropriate.

In order to verify if “staying at home had an impact on mortality rates,” it would be necessary to address many other issues in the analysis, including, but not limited to, omitted variable bias, measurement error, and endogeneity of the regressors. However, as shown above, even in a purely correlational analysis, with no causality claims, the applied methodology will simply deliver a weighted-average of coefficients across the two regions. An estimated coefficient $\beta \approx 0$ does not imply that there is no association between the variables in either country. Therefore, their conclusion does not follow from their regressions.

## Supplementary Information

Supplementary Information.
