# Supplementary material for: Pairwise difference regressions are just weighted averages
Source: Sci Rep. 2021 Nov 29;11:23044. doi: 10.1038/s41598-021-02096-3 (PMC8630001; doi:10.1038/s41598-021-02096-3)
Supplement: Supplementary file 1 — Supplementary Information. [file 41598_2021_2096_MOESM1_ESM.pdf]

Simulation

October 26, 2021

[1]:

```python
# -*- coding: utf-8 -*-
"""
Created on Tue Oct 26 10:27:47 2021

@author: Carlos
"""

from scipy import random
from scipy import stats
import pandas as pd
import numpy as np
import statsmodels.formula.api as smf

def simulation():
    T = 1000
    sigmax1 = 10
    sigmax2 = 10
    sigmaepsilon = 1
    beta1 = 10
    beta2 = -10

    x1 = random.normal(0,sigmax1, T)
    x2 = random.normal(0,sigmax2, T)
    epsilon1 = random.normal(0,sigmaepsilon, T)
    epsilon2 = random.normal(0,sigmaepsilon, T)

    y1 = beta1 * x1 + epsilon1
    y2 = beta2 * x2 + epsilon2

    y = y1 - y2
    x = x1 - x2

    DictFrame = {
        'y': y,
        'x': x,
        'y1': y1,
        'x1': x1,
```

```
        'y2': y2,
        'x1': x1
        }

    Frame = pd.DataFrame.from_dict(DictFrame)
    model = smf.ols("y ~ x - 1", data=Frame).fit()
    return model

params = []
for tries in range(0,10000):
    model = simulation()
    params.append(model.params[0])
```

[2]:

```
params
```

[2]:

```
[0.21696784412245484,
 0.05197357119058173,
 0.011451828438855692,
 0.07892072984562266,
 0.19963278738448315,
 0.21175732699123284,
 -0.5012324351548241,
 -0.2658177652544963,
 0.1024541519905309,
 0.10524207128044205,
 0.4527150976147602,
 -0.050909052886450 81,
 -0.1516355569808888,
 0.7443247109824462,
 -0.32422964504684726,
 0.5271526639961501,
 0.20332341676230312,
 0.12093472819427525,
 -0.45132008799210777,
 0.30073068861722085,
 0.12970266408047632,
 0.03775295722471164,
 -0.019278171676866254,
 -0.5951466104044679,
 0.196841192979263,
 -0.037105765993674114,
 0.1936525188888305,
 0.19105089603134678,
 0.36566590100537566,
 -0.26725029629369573,
 0.366209168920661,
 0.002672662506465834,
```

-0.059421810068755014,
0.13179668369787934,
0.17424641428214957,
-0.08884000262689434,
0.5072570705111752,
0.13217726106347816,
0.2522470699666477,
0.1005804664928038,
-0.12037842983619776,
0.6306626574248445,
0.0113417975588683,
-0.08312449247956347,
0.09136448641665834,
0.00653092414159448,
-0.47865724213535354,
0.2302105924679038,
0.1274054228446307,
0.041301417894494485,
-0.1506814209450947,
0.3993014953370888,
-0.009777005976988218,
0.3606801044963606,
-0.5053056308345651,
0.11601943192140776,
0.3270111302099133,
0.07036998199533581,
-0.9094620417455059,
0.18452933262283014,
0.24346413188128763,
-0.03274591316665336,
0.5328475611865755,
-0.07579594593380753,
0.2813144505726197,
-0.2605974613006302,
0.09870552552543352,
-0.2417704062430872,
-0.4677131980493184,
0.24273101846873188,
0.11711957181049326,
-0.11485931284502934,
-0.03974278482288752,
-0.3744394945880791,
0.24741027097231044,
0.04377046145525057,
0.20226258269942476,
-0.23618662179501862,
-0.5905146648788016,

-0.02449582925120293,
0.18048119805647034,
0.34021594649771836,
-0.06892780484195854,
0.304033510989372,
0.25470917746568555,
0.12174388875130399,
0.09490656166848738,
-0.20973413847264025,
-0.024824298216801655,
-0.33084439246315833,
0.3533639753783062,
-0.10006907546379887,
0.5580946657095258,
-0.029763736316187592,
-0.12617416120858496,
0.49969321025991986,
0.12070106115206358,
-0.1485044959172917,
0.3574470179189172,
0.4235896142291931,
-0.4483259849830693,
-0.011179703033555893,
-0.3926537876922878,
0.18563720288721058,
-0.08520717539467913,
0.1263518789777136,
-0.010166222798531577,
-0.4188136164544267,
0.23783003155719468,
-0.46963783510892754,
0.18931113608547342,
-0.16390588684060636,
-0.11225107692138331,
0.07717223491739802,
0.4161773703294359,
0.44581866848250656,
0.7458547502723699,
0.11293101722657295,
-0.3981916725324941,
-0.20561372656838428,
0.320993030231171,
0.18025073487333254,
0.254915697781585,
0.5659725835944428,
-0.26099750115873577,
-0.19010124184488392,

0.17421660555488072,
0.25655205332853537,
0.28187256997871596,
-0.4482146870615759,
-0.18021846200703118,
0.23721858954336628,
0.11051160508450397,
-0.1490031389251001,
0.15480315582063084,
0.5631374561565936,
0.13593814489762018,
0.037976171919364546,
-0.5061857104751998,
0.9005823786601614,
0.02223282728120124,
0.18396904448648815,
-0.21372261529440356,
-0.2728344089098081,
-0.3489389855073284,
-0.3044669377335551,
-0.3345822721523104,
0.07637219066575829,
0.27439212670375523,
0.3571616484481365,
0.4110845923387627,
0.0714034040912362,
-0.15467518819750103,
0.12451542992581746,
-0.4440661150539207,
0.40809428906414336,
0.03917289143835634,
-0.19655225875930032,
-0.12414149701144951,
-0.09070188304695863,
-0.14728215554521942,
-0.525668983500613,
-0.26199722454777946,
-0.36575508604660995,
0.21104242397768605,
-0.08719585519234263,
0.0425494111978893,
0.3660591334426242,
0.016298054281272928,
0.1116326356188175,
0.2657923793954758,
0.34661354856316207,
0.2777691746429456,

```
0.039737202469068755,
0.20518102784142023,
-0.12687673835878527,
0.07295288535617511,
0.34893487540372203,
-0.10227021778404533,
-0.14616158514323488,
-0.28192675342364276,
0.11840751892823533,
0.29552357878376917,
0.005139476441931967,
0.3120155613636816,
-0.042556109634000666,
0.4377204311076519,
-0.49981744482019963,
-0.4382862696632122,
-0.24876006555084315,
-0.25219711879336865,
-0.13474313461598594,
-0.3857853376705758,
-0.34343461319182933,
-0.057416778440707306,
0.20686837027640342,
-0.5567086264999347,
0.088474843880601,
-0.3204116297321499,
0.01672867023688719,
0.46028130419536906,
-0.43724692045432667,
-0.5303095642818709,
-0.03285191340981129,
0.36105451023031987,
0.018972090918426415,
-0.5448959255465251,
0.09256622886694499,
-0.09825866237230559,
0.06564968393127621,
0.13590036457468524,
0.36604426768194975,
-0.03898134532868566,
0.4288467397404541,
-0.06589261626077061,
0.35769157562476617,
-0.40544081640759055,
-0.033816500401066485,
0.07463876948328907,
-0.1650135359206248,
```

-0.04973710917256627,
-0.23163394896671913,
0.04562482793913769,
-0.24198954593917063,
-0.3280075197018174,
0.27776455451149884,
-0.25327150256909753,
0.03543297333333299,
0.12645752322657391,
0.1918586708627617,
-0.3737800691012961,
-0.15381521296017206,
0.3789753564302103,
0.12868467349271628,
0.14356308030414724,
0.07632534429053393,
0.8542629590152473,
-0.4706571112627713,
0.08963245710387965,
-0.14888589760798693,
-0.5291002609708331,
-0.07039066835472749,
0.0673705127395367,
-0.23103170079132684,
-0.34104551604138894,
-0.2271540095016278,
0.4687628986649639,
0.035775153595442644,
0.012291546208790957,
0.11004537063447856,
0.03189309414200997,
-0.07076790757892844,
-0.1424890794451379,
0.010864935510758393,
0.3433356586373809,
-0.28046102135614426,
-0.13924164774057396,
0.1403351887193251,
-0.5770198947192064,
-0.2178918285286581,
0.40523219341000666,
0.14412608648302705,
-0.5207994521774235,
-0.28726056043713144,
-0.2831250020648527,
-0.37118535271722275,
0.10902405469236467,

0.20156373812858835,
0.0021254463254012923,
-0.425994207558704,
0.24196821705368304,
-0.3305023564469822,
-0.0569777822747482,
-0.8977579483274221,
0.5815779480148853,
-0.8617079287303828,
0.27829514669302646,
-0.3123334172401375,
-0.6785533525352612,
-0.05938854869064121,
-0.5805390065449926,
0.036409466170492136,
-0.34812569741789223,
-0.6306549922290744,
-0.638308699458985,
-0.18425626160103198,
-0.33233860174027974,
-0.19890131443464165,
0.4513844079671111,
-0.06212990821695352,
0.0657812260453418,
-0.1805666611971251,
0.06149346053789326,
-0.1349154294652082,
0.13574472502603602,
-0.5234768428897303,
-0.07803071265412109,
0.5697842618317777,
-0.5288937378703578,
0.05469856694304982,
0.6867579165331483,
-0.2757724418770876,
-0.04830982084817809,
0.20425020361839874,
0.6755874928152588,
-0.4559366901901044,
0.38949041912864646,
-0.23407235724024444,
0.14889327521927367,
-0.23282219994318776,
0.21269524583609983,
0.13447626530925816,
0.7429165123303343,
0.3317987840679859,

0.34276558888285014,
-0.2521970894719261,
0.32047944736225054,
0.21724027946818045,
0.26440328805135543,
0.5252983158319563,
-0.15474189637650068,
-0.9371149477916858,
0.09952077067874458,
0.21313404857581608,
0.20839641595459568,
-0.6064376639961979,
-0.05037643155503041,
0.19394391057980936,
0.10882020412862373,
-0.2529073135705105,
-0.1709962500992159,
0.047009036381443575,
0.25380977403055116,
0.16933368033911078,
-0.13638906327657646,
0.008074166916752473,
0.17045186328873677,
0.08177432623331563,
0.04273188539232679,
0.24936410048858065,
0.23485072567214435,
0.012364305708276069,
0.16203505450746586,
-0.005840953837793955,
-0.47140560815245897,
-0.17789999118728422,
-0.010291688865445736,
-0.1896956946351211,
0.22399369834459448,
-0.09487590095068549,
0.08749491488837696,
0.2583988908038957,
0.06039863913896681,
-0.15120662183451017,
0.01542472374886017,
-0.24259578979007326,
-0.24959453458511843,
-0.326813280788869,
0.22565881016775083,
0.12893268152341292,
-0.26793896884979174,

-0.2586953579293749,
-0.33028300234645785,
-0.337741070289688,
-0.3304651320038642,
0.20006151780323814,
-0.0927315755122042,
0.25284408819409837,
-0.19650893565163519,
0.047201600421228396,
0.09598058822077563,
-0.2560621336671205,
-0.10648343177216896,
-0.5405867193540493,
-0.005618146232890489,
0.2747969896700601,
0.15525327158308466,
-0.39040937998184705,
0.3010000241641799,
0.28880673407261487,
-0.4136487800010616,
-0.051278454998417045,
1.1727619024005103,
0.21917422080096244,
-0.10857159018783201,
0.05171000007862653,
0.05406962332224366,
0.5157852512968168,
-0.22393911501129105,
-0.2307123050367647,
0.03375172526337758,
0.9356669995263001,
0.32400774563940915,
-0.08430249528183742,
0.42196659783218404,
0.11094547916164378,
-0.2655224701126993,
0.18861534534627888,
-0.649465095687604,
0.4566474481572329,
-0.14444679256617152,
-0.0031700459751643484,
-0.43199227601365164,
-0.01378554498857236,
-0.06807761456281625,
0.011612450915223754,
0.011232639808125208,
-0.38678664534333784,

0.36564723070995525,
-0.4883281852620064,
-0.686627080915492,
0.23323301914024555,
-0.07249983465808091,
-0.18878838377590335,
-0.05132509254459895,
-0.775756379965358,
0.2084399910551682,
-0.28928341318480727,
-0.27567989986827734,
-0.818203203310561,
-0.3882500765920001,
-0.28231974766039225,
-0.0004451091038430999,
-0.07060411919240377,
0.08830447515709705,
-0.3335256114434425,
0.27645336096749107,
0.2077274725636001,
0.0899307391383937,
-0.1955067815109469,
-0.4254937999850885,
-0.25854692336849333,
-0.2926304920522782,
-0.1514874141379972,
0.6821221605427275,
0.2637197694695519,
0.34117901608577783,
-0.4629305775856377,
-0.8073296609646062,
-0.36427272841408687,
0.06613856466597781,
-0.3270090585281221,
-0.2117999237809351,
-0.8676019053400443,
-0.4217232982740585,
0.21515200570052795,
0.07198721730653473,
0.38734600094642024,
-0.09275239785826311,
-0.0253669789224239,
0.01251195130203786,
-0.6779921643451055,
-0.031815531302014616,
0.23098510841502157,
0.36500154787909556,

0.3143320470477231,
0.271291712118091,
-0.39970026010131365,
-0.18029299563416723,
-0.21730838519617102,
0.05928119903345497,
0.1700426029514664,
0.06872468052265678,
-0.3625350811943779,
0.45646895718282465,
-0.4785872715796716,
-0.5135475422865159,
-0.016542916656261356,
-0.44999773435514423,
-0.30387924866001226,
-0.25465789496462926,
0.0027293907934535233,
-0.24258119705887055,
0.3689037124524963,
0.38312332935076887,
-0.5850476345065073,
-0.5969335492050316,
0.020947249912938642,
-0.09774537738735589,
-0.3082615089533661,
0.16809242718098297,
-0.21117129768920523,
0.029023979477051765,
0.2608408156919793,
0.40872313795978976,
-0.3356441541431686,
0.49831422255567814,
-0.004243689584365509,
-0.4737589379911622,
0.44414724673324285,
-0.06883259387598617,
-0.5319087349620355,
0.030762742132939835,
0.04662587311532074,
0.4594682443583454,
0.06321238941357672,
0.4182706835858546,
0.0699397234363946,
-0.10880144287294079,
0.11832981076614713,
0.07841978463418331,
-0.21575854356231472,

-0.1440918284599925,
0.217243511183119,
0.32163932380381965,
0.29757697128006955,
0.10151980705055194,
-0.332285336015135,
-1.0467291705248596,
0.2629542832494911,
-0.06844915039021723,
-0.4977731780621494,
0.4340618671690714,
0.20813234446404286,
-0.19867437005519392,
-0.19504037565082027,
0.056238128978173596,
0.30385472444993916,
-0.3757386872681415,
0.1896216187641665,
0.41946986815005594,
0.25816020058798383,
-0.0238156430687443,
-0.1271322830091856,
0.3419881131767528,
0.5910392486067365,
0.1244582926961558,
0.22509702077313748,
0.062348888221914214,
-0.2361864918461929,
-0.17061011285656175,
0.25525076272651304,
-0.19059793476017906,
-0.05279683741867619,
-0.10583023175871464,
-0.6311501494513128,
-0.13721120871499481,
0.27761843200892705,
0.015233980755694848,
0.18640248293658673,
0.4035533988054041,
-0.5119672053316662,
0.29998097562170334,
0.01772543320493436,
0.3559925716169572,
-0.041354328270821666,
-0.11505330080838577,
0.4007914807761545,
-0.5063332119918569,

```
-0.6229448773777551,
-0.033984544655332814,
-0.08550058358463064,
-0.4404565341023195,
-0.2773800131749057,
-0.27146892763443875,
-0.017734319536990062,
-0.12078705782801005,
0.15536240219807007,
-0.19010914670646112,
0.6043577638199515,
-0.28596571575571883,
0.00011863781686551056,
0.20547460719613098,
0.7019811226201222,
-0.41679118638822876,
0.2735713817678261,
-0.1266953350965676,
0.5444023541773945,
0.1948907441600698,
-0.3414774191430502,
-0.12738160050799616,
-0.85326158784649,
0.5099423422901436,
-0.2509963751047817,
0.059648201608493934,
-0.49537587618101997,
0.4474912033681586,
-0.5087874567718969,
-0.14480085444357757,
-0.2247165758847125,
0.13751281529918977,
0.3274795067210567,
0.722863552790652,
0.31404533484993813,
-0.06621223626555123,
-0.11423374443950243,
0.04306240161727168,
0.894220985715981,
-0.08604991602540261,
0.31450761574740954,
0.07388470681494322,
-0.07874734164381275,
-0.24394744112515865,
-0.2651189226903977,
0.3825919473504102,
-0.07700032591251908,
```

-0.024743347531032397,
-0.005524436432408936,
-0.5479496104044755,
-0.011088646418195715,
-0.4059633674911932,
-0.7495766695653416,
0.3249994666901657,
-0.29687143212764466,
-0.27870457010810656,
0.3931874046486016,
0.3033796513110657,
-0.039094065796046205,
-0.12705444626978515,
0.1829690650712615,
0.5172722859183277,
-0.28941901388061186,
0.4675677949076159,
-0.0094618329645971,
0.16986809073770348,
0.14952925532234118,
0.30095886989576315,
-0.5942776496294346,
-0.30814271420381073,
0.055289298901495726,
0.6310102464589209,
-0.13438747029887044,
0.11697644611049918,
0.17054008830061795,
-0.8080571523525917,
-0.21752775405670002,
0.009403257414304783,
-0.141912206623123,
-0.3476400974111778,
-0.1142880782090957,
-0.002842040656921299,
-0.2094379797318299,
0.4858602164189521,
0.13346415854960342,
0.37992334115800463,
0.22495034774877865,
0.267505705872831,
-0.016604366284106026,
0.5277481738964783,
0.5989121924773433,
0.2678805942528519,
0.531671682283541,
-0.4124423028602029,

```
0.028736433123275416,
0.31281359245421203,
0.1276060525886201,
-0.6132463923311096,
-0.23618686285159798,
0.13622363472503188,
-0.5373378990789959,
0.23175716515560862,
0.35782901904460196,
-0.14609651776495636,
-0.49783488127759623,
-0.19492223551551907,
0.06524507158752066,
0.4131660763306379,
-0.6668681566128551,
-0.4924437748894991,
0.2716255236341486,
0.1584379281078838,
-0.5155439387483616,
0.07913992786503776,
-0.14425271755829783,
-0.0816821309338143,
0.27738896605653635,
-0.5493035717899855,
-0.07515624167314411,
-0.0600852027415735,
0.050928099606481175,
0.33191350752132964,
0.4048478429255751,
0.4307610644871742,
-0.05104678789364265,
0.08681002542342,
-0.14318792333022873,
-0.2929552788900054,
-0.01910135844682362,
-0.10690768367852896,
0.05243372859246062,
0.02842191425959173,
0.03441594049608631,
-0.3630203295001736,
-0.35378569806737253,
-0.06083252322445833,
0.26711478574753456,
-0.03651702220274142,
0.21435199321823162,
-0.05283426364075734,
0.1369266861184339,
```

-0.5249417536247138,
0.12554548032401805,
-0.2062857209921085,
-0.03747314326362838,
-0.3262176199609472,
0.0029758034623598717,
-0.19412476907651827,
-0.5243341115197464,
0.20117399243067424,
-0.0864024046757178,
0.1399664510175782,
-0.1760420709410731,
0.6979601240936726,
0.051705380308665005,
-0.38382487585325337,
0.06630638587107027,
-0.5669747770126041,
-0.6104368967923641,
-0.18738743671075347,
-0.08210970995127334,
-0.16028886554741334,
-0.4640914266087627,
0.16013934541463531,
-0.3381468023075675,
-0.5212881073608526,
0.23627242913175184,
-0.2994202190775802,
-0.19645569518680756,
0.6253738007542553,
0.09934828511693393,
-0.1726982696572687,
-0.2770505300334892,
0.022705482827180823,
-0.18281314360421372,
0.49800588263742,
-0.03671620501694611,
-0.05446982868516759,
-0.1987757181415683,
-0.1943306588009167,
-0.01915716647099147,
0.35570630050511554,
0.3475077141388777,
-0.08765778674856389,
0.6785060863611692,
0.2338826882606696,
-0.2453911830423423,
-0.06202254692402713,

-0.22092757728725604,
0.2116027811521275,
0.1383289514268408,
0.022210565865508097,
-0.21359584831307235,
-0.28102579893827895,
0.5714130118153489,
0.06405858562548206,
-0.2811481708989455,
0.399577350251217,
0.15698206918139157,
0.06856835516268994,
-0.12564824932988705,
0.38043214929330554,
0.3038263097686722,
0.5730334761621351,
0.2509131697882564,
0.19852977519947257,
-0.014660084269279089,
0.5788318566019617,
-0.05674831154359132,
0.17134621712754836,
0.5059452986477465,
0.02897293025165476,
-0.3320722269826471,
0.3048020033132717,
-0.043616388951436214,
0.14789948151162696,
0.2830925906014582,
0.41922143316886384,
-0.699888208367376,
0.4518683636189446,
-0.815981181895682,
0.013546186437310231,
-0.5718303582417683,
0.05449633167631324,
0.005889462505677193,
-0.16473021579926528,
-0.5935971091231783,
0.14148432433321478,
0.7622570826007087,
-0.5045005537203574,
0.14200797181791674,
0.22353415704991558,
0.21804650936816056,
-0.3025211539469426,
0.009835962240461904,

-0.11412104020423634,
-0.27995842184581365,
-0.28234014240214117,
0.018231672605270477,
0.18820717354627545,
-0.258898650398786,
-0.06634445071607956,
0.6002259181317776,
0.2020810777964243,
0.15860654493206863,
-0.5435069671776053,
-0.37996303503351336,
-0.2601761691155079,
0.043948275312804014,
0.35325337821103986,
-0.3899315968027218,
0.523964830942883,
0.3488123102435344,
0.26658262985359243,
0.09437653852126221,
0.49415159172307066,
-0.5355766289915114,
-0.12162899437322727,
0.300421879678721,
-0.31043059803799644,
0.05296320305946181,
0.008411405956118986,
0.5114526342919652,
0.13278061222573528,
-0.5933943968538902,
-0.3932861733990364,
-0.11521927262806944,
0.1571322548661706,
0.2885861912194876,
-0.40568089857695233,
0.04441265935115099,
0.20938673797411272,
0.258940493106435,
0.04203174589097053,
-0.45775755929576567,
-0.04171192686421048,
-0.40553423654701476,
-0.06706628208265089,
0.07789158799746099,
0.14964923930466556,
0.025862281770072923,
-0.3947013262468799,

0.48854720083724984,
0.0471727708281552,
-0.18645016368394074,
0.5254324330781088,
-0.2293179991546076,
0.10782012705133118,
-0.4547522308617659,
-0.4779580119510909,
-0.5600777478006086,
-0.3486039317838403,
-0.3045495543780614,
0.4769840805570246,
-0.05496388129257153,
-0.662674710062888,
0.6020346504896524,
-0.38916835094478774,
-0.0012971688604785855,
0.21785976982608485,
-0.30408868860910454,
0.07863540415600895,
0.40944800501314405,
0.10208049666914669,
-0.26872608568264644,
-0.43912049059684277,
0.3558286917968094,
0.2739873299309572,
0.42546150658542287,
-0.4844703049844945,
0.010740037586083911,
-0.5791089259657317,
0.008974218988381147,
-0.17653977434039414,
-0.38187459026585124,
0.2519678085796542,
0.4612721281456768,
-0.6374781649515153,
-0.0367215957099868,
0.06614043380766221,
-0.18314806565015115,
0.12380827981454359,
-0.11069816652926961,
0.6372592968337962,
0.10204463921891929,
-0.3713007664277401,
-0.12912372362218583,
0.3809094644418629,
0.11609849980530992,

-0.2667491701827571,
0.09788611038252368,
0.07068606678701772,
0.19326878158585653,
0.40845610954188105,
0.2387352833104263,
0.17514045475911524,
-0.15286373976966552,
0.35168086323453374,
0.136222080880025,
0.14729934205722078,
-0.04533308422096727,
0.036017081841232645,
0.2590809932521557,
-0.05324451156175196,
-0.39283505366581384,
0.2762522016125709,
0.1665876448296079,
0.5554431885981435,
0.12468351887349272,
0.48249403015244535,
0.36324644039324716,
0.17327981447348662,
-0.3943249674241902,
-0.2740807508598776,
0.07214301521948058,
-0.17485975461934478,
-0.23632188044315605,
-0.15778011790446975,
0.06118091165836396,
0.17077502270783432,
-0.17868388034065444,
-0.18550364320160428,
0.35722934350485797,
-0.5987849896853692,
-0.14637391265178698,
0.46245192298740484,
0.2989108991536589,
-0.07563498896201888,
-0.26055138723518145,
0.04788121911847451,
-0.1621799236266336,
-0.0862276217072771,
0.06066882559191628,
0.4247895794297567,
0.007169979917400121,
0.1086165088888803,

-0.18018955697909195,
-0.28893563986054327,
0.02047586727304017,
0.06678002540175357,
-0.1879892380985038,
-0.4503709027438266,
-0.2520131826681891,
0.6034122036828486,
-0.19097506815163734,
0.10704605907164691,
0.5352600131310148,
-0.29064195735442855,
-0.018902414313581917,
0.19663529943685698,
0.22149506820751436,
-0.0347399918784789,
0.006894011350287638,
0.041061491337224726,
0.35238912280893636,
-0.18490492491936,
0.1170572491258303,
0.28890402636777535,
-0.07190322902141093,
-0.3990800794250046,
-0.3118361563090859,
-0.42161319109488465,
0.2817151200306454,
-0.5560789978127259,
-0.2828913587691636,
0.6276042137299747,
0.1287553770902297,
-0.7359841195166157,
-0.03149738786694953,
-0.18953737848419466,
0.04017079810858995,
0.4034710183251755,
0.07255742031536558,
-0.05004291142126621,
0.26253641483719503,
-0.5477383652855525,
-0.36710208055530813,
-0.23044384057400583,
-0.25326389474380107,
-0.36394126052480436,
-0.009934968956363477,
0.011326663616217689,
-0.026797562533033427,

```
-0.048926098353765185,
0.6195107076687241,
0.4831584080398293,
-0.09580159194833548,
0.37814229885928974,
-0.5270389968541955,
0.3467457910717677,
-0.3407891044855201,
-0.22451566249650695,
0.17425223246077065,
-0.003103493207286395,
0.20843722968284623,
0.24938634880692398,
-0.13692113210645923,
0.11720419424516447,
-0.24695825644868885,
0.18899132481509326,
0.259949377582506,
0.21944516480710424,
-0.405669211187471,
-0.1099228184379906,
-0.8523775596787895,
-0.06806871929306439,
0.02976760949594323,
-0.45328031163713683,
-0.1168540459189607,
0.17118345463004997,
0.12935806857150672,
…]
```

[3]:
```
import matplotlib.pyplot as plt

fig = plt.figure()
plt.hist(params, bins=250)
plt.axvline(0, color='black')
plt.show()
```

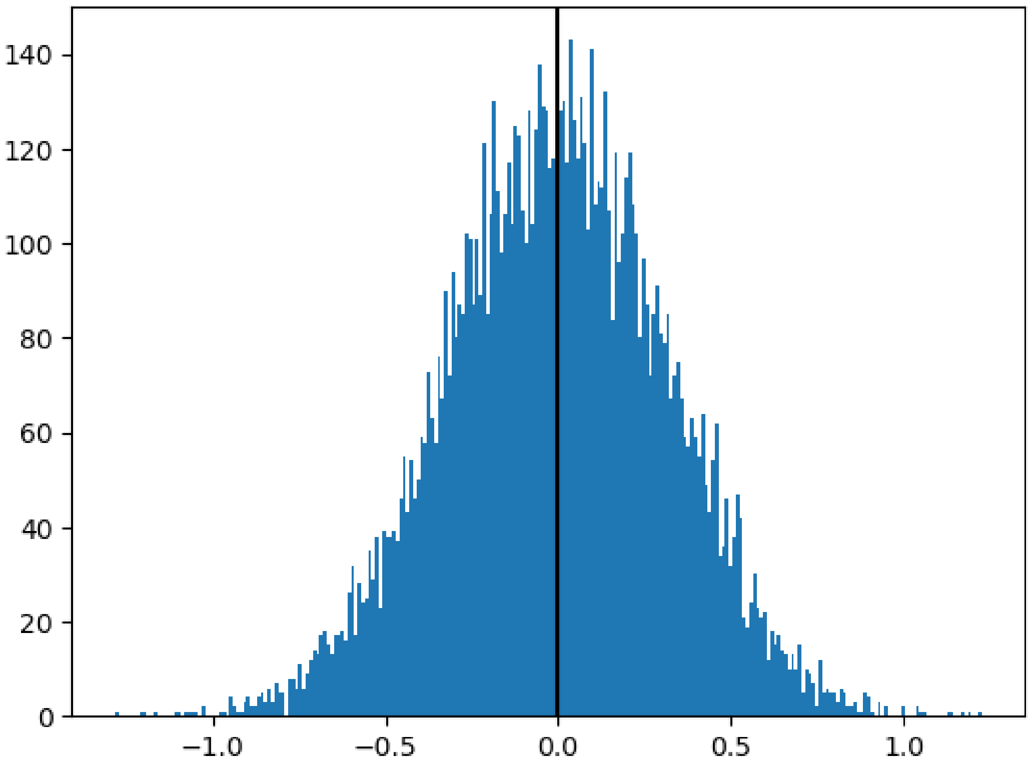
140
120
100
80
60
40
20
0
−1.0
−0.5
0.0
0.5
1.0

[ ]:
